# Correlating Mid‐Infrared Absorption With Radiative Efficiency in Passive Daytime Radiative Cooling Systems

**DOI:** 10.1002/marc.70329

**Published:** 2026-06-17

**Authors:** Emma Spotorno, Paola Stagnaro, Lucia Conzatti, Simona Losio, Roberto Utzeri, Francesco Bisio

**Affiliations:** ^1^ OptMatLab, Dipartimento di Fisica Università di Genova Genova Italy; ^2^ Istituto di Scienze e Tecnologie Chimiche Giulio Natta, Consiglio Nazionale delle Ricerche Genova Italy; ^3^ Istituto di Scienze e Tecnologie Chimiche Giulio Natta, Consiglio Nazionale delle Ricerche Milano Italy; ^4^ Istituto Superconduttori Materiali Innovativi e Dispositivi, Consiglio Nazionale delle Ricerche Genova Italy

**Keywords:** optical properties, polymer composites, radiative cooling

## Abstract

Passive daytime radiative coolers (PDRCs) are physical systems capable, on the Earth's surface, of achieving a net irradiation of energy toward outer space even under direct solar illumination. In order to achieve this amazing feat, PDRCs require tailored photonic properties all the way from the near‐ultraviolet to the mid‐infrared (mid‐IR). In particular, PDRCs require high reflectivity over the solar‐radiation range and selective absorbance (or equivalently emittance) in the mid‐IR atmospheric transparency window. In this study we fabricated PDRCs composed of silica‐loaded poly(4‐methyl‐1‐pentene) thin films hot‐pressed onto Ag‐coated stainless‐steel slabs, and we correlated the photonic response of these systems with their thermodynamic radiative efficiency. We clearly show that the presence of a strong emittance across the mid‐IR range corresponds to a higher radiative power around room temperature, thereby establishing a clear quantitative criterion for PDRC assessment.

## Graphical abstract

We establish a correlation between the mid‐IR photonic response of Passive Daytime Radiative Cooling systems and their thermal radiation performances, characterized within a dedicated setup that simulates the irradiance toward outer space under controlled and reproducible conditions. An apparent correlation is shown between their mid‐IR emissivity and their self‐cooling capability.

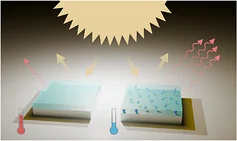

## 1 Introduction

Passive daytime radiative cooling (PDRC) is a disruptive technology that allows a physical system on the Earth's surface to reduce its temperature, even under full solar illumination, by reflecting the whole solar spectrum and simultaneously emitting thermal radiation toward the cold outer space [1, 2, 3, 4].

This effect is based on the ability of a material to show high thermal emission in the mid‐infrared (mid‐IR) atmospheric transparency window between 760 and 1250 ${\mathrm{cm}}^{-1}$, while minimizing unwanted absorption both in the solar‐spectrum and in the atmospheric scattering range, by exploiting a highly reflective surface and using materials that are transparent all across the ultraviolet, visible and near‐IR range [5]. Since PDRC does not require external energy input to achieve net energy radiation to outer space, it has attracted increasing attention for applications such as energy saving, thermal management, and sustainable cooling systems [6, 7, 8, 9, 10, 11, 12, 13, 14]. The performance of a PDRC system strongly depends on the optical and radiative properties of all the materials employed [9, 10, 11, 12, 13, 14, 15]. In particular, a high emissivity in the mid‐IR combined with suitable reflective layers in the visible range, is essential to enhance the cooling effect: multilayer structures composed of metallic substrates topped by polymeric coatings are widely investigated to optimize radiative emission [16, 17, 18].

The design of PDRC systems typically follows a layered approach. Above a supporting substrate, high‐reflectivity films are deposited, Al and Ag being the most‐widely employed materials [2]. This ensures the reflection of a large fraction of solar radiation, typically exceeding 90%–95%. On top of the reflective layers, PDRCs include the so‐called radiative layer (or IR layer): this layer is ideally characterized by optical transparency across the whole solar spectrum and over the atmosphere‐opacity ranges, while exhibiting selective absorbance (hence emittance) in the atmospheric IR transparency window. IR layers can be realized by single‐ or multi‐layers of inorganic materials (e.g. ${\mathrm{SiO}}_{2}$, ${\mathrm{HfO}}_{2}$) [2] or by appropriately synthesized polymers and composites [16, 17, 18].

The main goal of this study was to establish a correlation between the mid‐IR photonic response of the systems, here extracted by spectroscopic ellipsometry (SE) measurements, and their thermal radiation performances, characterized by means of temperature measurements within a dedicated home‐built setup that simulates the irradiance toward outer space under controlled conditions [19].

To this end, PDRC systems with variable amount of mid‐IR emissivity were fabricated and compared, showing an apparent correlation between their mid‐IR emissivity and their radiation‐mediated self‐cooling capability.

## 2 Results and Discussion

### 2.1 Sample Structure

Herein, an efficient radiative cooling effect based on the use of a polymer‐based layer with selective IR emissivity in the 670–1250 ${\mathrm{cm}}^{-1}$ range was obtained. Our PDRC systems were based upon a polished stainless‐steel slab covered by an optically thick silver layer, acting as mechanical support and highly‐reflective substrate. On the top of the metallic substrate, poly(4‐methyl‐1‐pentene) (PMP) films were hot‐pressed and acted as the emissive polymeric layer [20]. PDRC systems were prepared with PMP‐based films either in their pure form (henceforth referred to as pure PMP) or with embedded silica microspheres, dispersed into the polymer matrix to modulate the emissivity in the targeted mid‐IR region [18, 21, 22]. The average reflectivity over the solar spectrum was above 0.95.

The polymer‐based materials here explored are composites containing 6% by volume (vol.%) of micrometer‐sized silica (${\mathrm{SiO}}_{2}$) particles, randomly distributed in poly(4‐methyl‐1‐pentene), chosen as a matrix due to its high optical transparency over the solar spectrum. Indeed, isotactic PMP displays a unique combination of physical properties due to its distinctive molecular structure [23], characterized by the bulky iso‐butyl side branches, which confers it very low density (0.83–0.84 g ${\mathrm{cm}}^{-3}$) and high gas permeability [24]. Due to its successful employment in demanding applications, such as automotive components, high‐temperature resistant cling films, baking trays, and microwave‐safe containers, PMP is expected to ensure long‐term stability in the PDCR applications here discussed. Although PMP is a semicrystalline polymer, it exhibits excellent transparency (Haze ≤5%) and solar‐spectrum transmittance. Moreover, it exhibits a very low water absorbance and high chemical resistance, which ensures excellent durability with respect to environment exposure. The commercial PMP grade here employed has a $M_{w}$ equal to 520 600 g ${\mathrm{mol}}^{-1}$ and a polydispersity of 3.6, referred to polystyrene standards (see the Supporting Information). Different PMP samples have been loaded with either different monodisperse ${\mathrm{SiO}}_{2}$ microparticles with 10 µm (Si‐10), 5 µm (Si‐5), and 1 µm (Si‐1) diameter, respectively, or with a combination of the three in order to tune the optical properties ot the polymer‐based IR‐emissive layer. The inclusion of ${\mathrm{SiO}}_{2}$ particles has indeed the combined effect of introducing material with intrinsic optical absorbance/emittance in the mid‐IR atmospheric transparency window and to benefit from finite‐size dielectric resonances of the microspheres, that enhance their absorbance in the same spectral window [18, 25].

PMP‐based composites were prepared by melt mixing and extrusion carried out in a mini twin‐screw extruder. The thermal stability of the ensuing materials was assessed by thermogravimetric analysis (TGA) and their thermal behavior was evaluated by differential scanning calorimetry (DSC), as described in the Experimental Section. The obtained thermal parameters are collected in Table 1; data related to neat PMP (as purchased commercial polymer) and neat polymer after treatment in the mixer‐extruder (pure‐PMP) are reported as well for the sake of comparison. The other samples reported in Table 1 with their thermal parameters are labeled according to the size distribution of the incorporated silica microspheres. Sample Si‐10 contains monodisperse 10 µm silica microspheres, sample Si‐5 contains monodisperse 5 µm silica microspheres, while samples Si‐10/Si‐1 A and Si‐10/Si‐1 B contain distributions of 10 and 1 µm silica microspheres with volume ratios of 75:25 and 50:50, respectively. This study showed that the adopted melt blending conditions and the ${\mathrm{SiO}}_{2}$ incorporation do not practically affect the characteristic thermal transitions of the PMP matrix. This represents an advantage for the future expansion of the envisaged systems, since the same processing parameters and conditions typically used for neat PMP will be easily transferred to these PMP‐based composite materials.

**TABLE 1:** Thermal parameters obtained from DSC analysis for neat PMP and ${\mathrm{SiO}}_{2}$‐loaded PMP samples. Values of melting and crystallization enthalpy of composites are normalized with respect to the polymer amount and using 2.0 g ${\mathrm{cm}}^{-3}$ as the density of the silica microspheres.

Sample	${\mathrm{SiO}}_{2}$ type	${\mathrm{SiO}}_{2}$ composition	$T_{onset}$	$T_{vmax}$	I heating		Cooling		II heating	
					$T_{m}$ ( 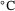 )	$\Delta H_{m}$ (J $g^{-1}$)	$T_{c}$ ( 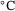 )	$\Delta H_{c}$ (J $g^{-1}$)	$T_{m}$ ( 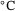 )	$\Delta H_{m}$ (J $g^{-1}$)
neat PMP	—	—	—	—	234	31	207	32	233	37
pure‐PMP	—	—	445	471	234	35	209	37	230	39
Si‐10	Si‐10	100	446	472	233	35	210	36	230	38
Si‐5	Si‐5	100	445	470	234	35	209	36	230	39
Si‐10/Si‐1 A	Si‐10/Si‐1	75:25	445	471	235	34	209	38	231	37
Si‐10/Si‐1 B	Si‐10/Si‐1	50:50	447	471	234	34	209	37	230	37

The morphology of the PMP‐based composites was analyzed by scanning electron microscopy (SEM) on cryo‐fractured cross‐section surfaces. Figure 1 shows the SEM images for Si‐10, Si‐10/Si‐1 A, Si‐10/Si‐1 B and Si‐5 samples. The silica microspheres are homogeneously distributed and evenly dispersed within the PMP matrix. See also the Supporting Information where SEM micrographs taken at different magnifications are shown.

**FIGURE 1:**
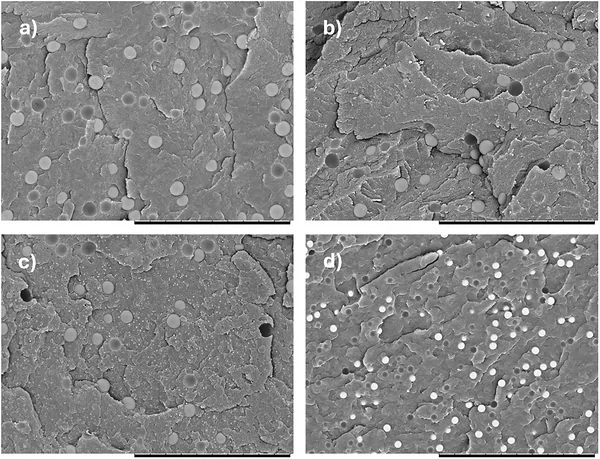
SEM images of the PMP‐based composite samples with different silica microsphere compositions. (a) Polymer matrix containing silica microspheres with a nominal diameter of 10 µm (Si‐10). (b) Polymer matrix containing a distribution of silica microspheres with a 75:25 volume ratio of 10 and 1 µm particles (Si‐10/Si‐1 A). (c) Polymer matrix containing a distribution of silica microspheres with 50:50 volume ratio of 10 and 1 µm particles (Si‐10/Si‐1 B). d)Polymer matrix containing silica microspheres with a nominal diameter of 5 µm (Si‐5). (SCALE BAR: 100 µm).

The polymer and composite films were hot‐pressed onto Ag‐coated stainless‐steel polished slabs, fabricated as detailed in the Section 4 and in the Supporting Information.

### 2.2 Optical Response

The experimental infrared‐SE response of the investigated samples was analyzed to examine differences in their absorption‐related spectral features. The discussion mainly focuses on the comparison between two specific PDRC samples, representative of different radiative behaviors: the pure‐PMP, and the one with the PMP film loaded with the 10 µm‐diameter spheres (Si‐10). The complete set of spectra of all the samples fabricated in the course of this study are reported in the Supporting Information. In Figure 2, the ellipsometry spectra of the two above‐mentioned samples are reported.

**FIGURE 2:**
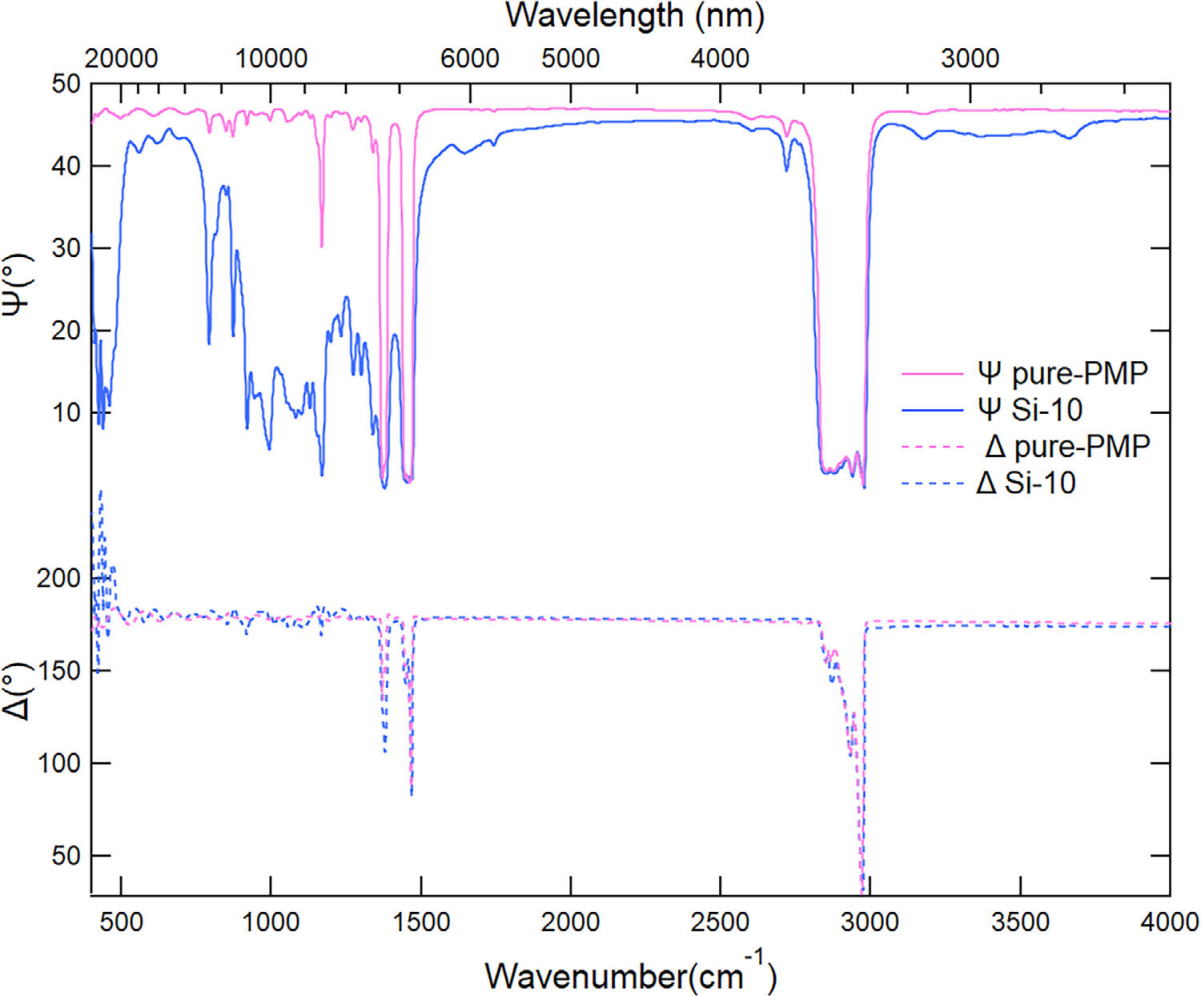
Ellipsometric parameters Ψ (solid lines) and Δ (dashed lines) for the pure‐PMP sample (pink) and the Si‐10 sample (blue), measured in the spectral region between 400 and 4000 ${\mathrm{cm}}^{-1}$.

Figure 2 compares the Ψ (continuous lines) and Δ (dashed lines) spectra of the pure‐PMP sample (pink curves) and the Si‐10 sample (blue curves). The Ψ spectra can be viewed as a plateau at around Ψ = 45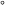 characterized by a series of more‐or‐less pronounced dips. Analogously, the Δ spectra can be viewed as a plateau at $\Delta \approx 180^{\circ }$ with dips in correspondence of the Ψ dips. It is important to note that Ψ and Δ reflect different contributions to the optical response: variations in Ψ are more strongly influenced by absorption effects, while Δ is generally less sensitive to them. In the mid‐IR, the pure‐PMP shows only weak and poorly structured features in both Ψ and Δ, while the Si‐10 sample shows a richer set of spectral features, especially in Ψ. Both samples show nearly‐identical spectral features around 1400 ${\mathrm{cm}}^{-1}$ and 3000 ${\mathrm{cm}}^{-1}$, typical of the vibrational fingerprint of the PMP polymer [26].

The Ψ, Δ values of the samples are a consequence of the whole sample structure (i.e., metallic substrate coated with polymeric films), and their interpretation typically requires a detailed modeling of the dielectric properties of all the materials involved [27, 28, 29, 30]. For the purposes of this study, however, a semi‐quantitative interpretation is sufficient.

To this end, we observe that the apparent dips in Ψ with respect to the $∼45^{\circ }$ upper level are a signature of optical absorbance in the polymer film. The depth of the dips is proportional to the first order to the corresponding absorbance in the polymer. It is therefore clear that introducing the function $A_{eff}$, defined as:

(1) $$A_{eff}=\Psi_{max}-\Psi$$

where $\Psi_{max}$ is the maximum value of Ψ, allows to obtain a first‐order estimation of the spectral optical absorbance of the polymer layer. To this end, the reader is referred to the Supporting Information for a detailed justification of the correspondence between the Ψ dips and the optical absorbance of the polymer layer.

Once the absorbance (or analogously, the emittance) of the polymeric layer is ascertained, its temperature‐dependent radiative efficiency must be determined. To do so, the effective absorbance $A_{eff}$ must be multiplied by the appropriate blackbody radiation spectrum B(ω,T) (orange curve in the top panel of Figure 3) evaluated at the foreseen operating system temperature, typically around T=300 K when on Earth's surface. The B(ω,T) has a broad maximum centered at around 800 ${\mathrm{cm}}^{-1}$, and decays to almost zero around 2500 ${\mathrm{cm}}^{-1}$. This implies that absorption features in the 500–1500 ${\mathrm{cm}}^{-1}$ range will be effective at radiating at 300 K, while spectral features located out of this range will not contribute significantly to the system's radiation.

**FIGURE 3:**
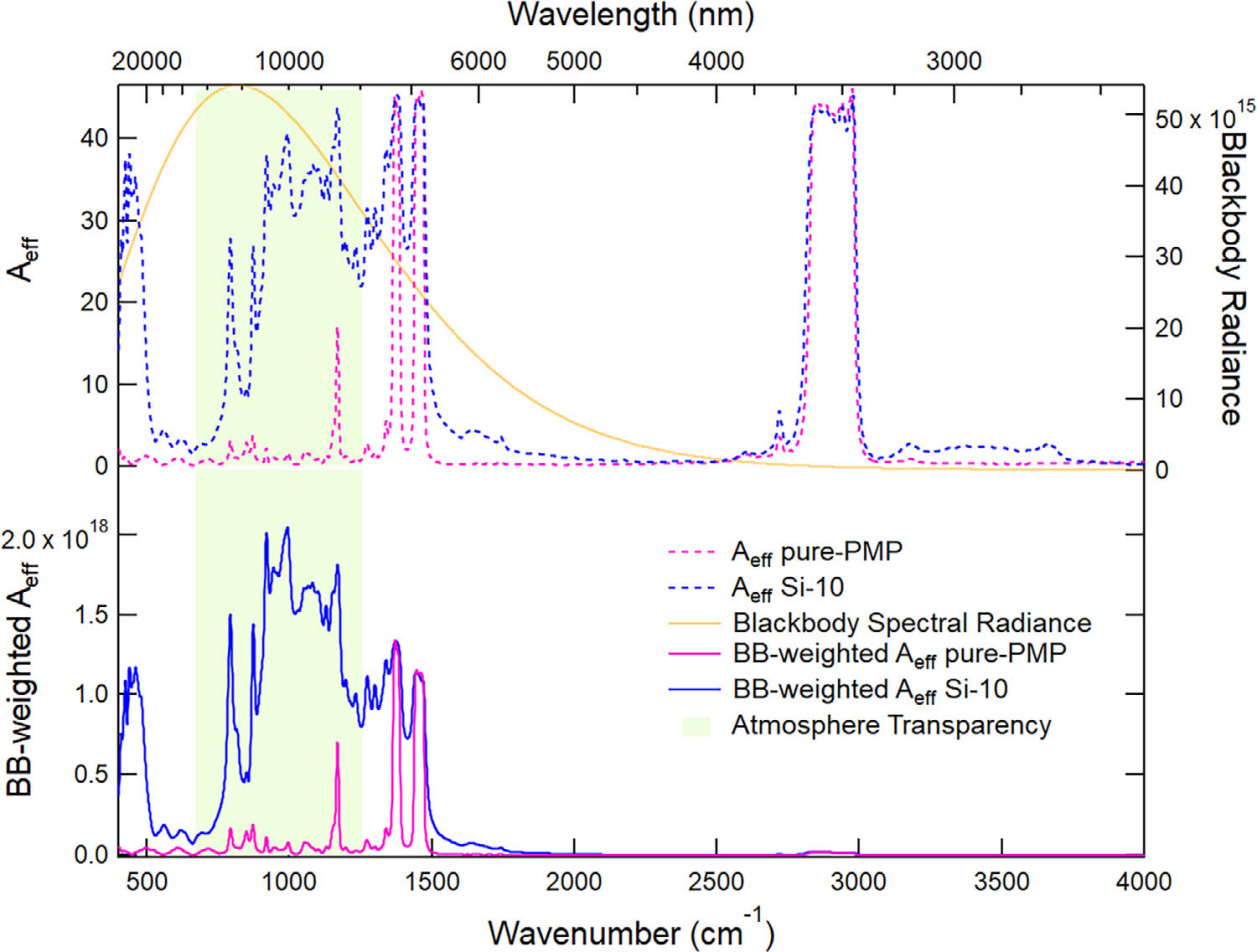
$A_{eff}$ spectra of the investigated samples. The upper panel shows the unweighted $A_{eff}$ spectra (dashed curves) of the two considered samples together with the blackbody emission spectrum (light‐orange curve). The lower panel reports the corresponding blackbody‐weighted $A_{eff}$ spectra (solid curves). The green‐shaded region represents the spectral extension of the mid‐IR atmospheric transparency window.

In the Figure 3, in the top panel, we report the $A_{eff}$ spectra of the pure‐PMP and the Si‐10 samples (pink and blue curves) along with the B(ω,T=300K) spectrum (light‐orange curve). In the bottom panel, we report the $A_{eff}$ spectra weighted by the B(ω,T=300K) curve. The green‐shaded window represents the mid‐IR atmospheric transparency window. The absorption bands around 1400 ${\mathrm{cm}}^{-1}$ and 3000 ${\mathrm{cm}}^{-1}$ lie outside such transparency window, leading to a potential radiation absorption by atmospheric scattering, that should however not invalidate the PDRC effect [18].

In the weighted curves, we can notice that the strong absorption bands around 3000 ${\mathrm{cm}}^{-1}$ of the two samples, apparent in the upper panel of Figure 3, do not contribute to energy dissipation by radiative emission at T = 300 K, whereas the absorption in the atmospheric‐transparency window are spectrally matched with the blackbody spectrum.

Furthermore, the weighted spectral response of the pure‐PMP sample shows lower radiative efficiency in the atmospheric transparency window with respect to the Si‐10 sample. Compared to pure‐PMP, the inclusion of microspheres therefore promotes a spectral response that is more suitable for radiative cooling applications.

Whilst this blackbody‐weighted analysis does not provide a quantitative measure of emissivity or radiative power, nonetheless it highlights differences in the relative spectral emission efficiency of the samples, providing a basis for evaluating their spectral suitability for radiative cooling applications. We therefore move on to characterize their thermodynamic response.

### 2.3 Thermodynamic Characterization

The irradiance efficiency is assessed measuring the time evolution of the temperature difference ΔT occurring between the PDRC samples and a reference low‐irradiance dummy (here Ag‐coated stainless steel), under high‐vacuum conditions, when both are placed in front of of a cold surface, as described in detail in Section 4. Figure 4 shows the temporal evolution of the temperature difference ΔT, defined as $\Delta T=T_{dummy}-T_{PDRC}$ for the pure‐PMP sample (crosses) and the Si‐10 sample (circles). A positive ΔT implies a lower temperature of the PDRC with respect to the dummy. The origin of the time axis is defined as the instant in which ${\mathrm{LN}}_{2}$ @ 77 K is poured into the cold sink. The marker color indicates the PDRC temperature during the process, according to the color scale on the right of the graph. The isolated markers at a time delay of approximately $2\cdot 10^{3}$ s correspond to the maximum ΔT achieved for samples Si‐10/Si‐1 A (diamond), Si‐10/Si‐1 B (triangle) and Si‐5 (pentagon).

**FIGURE 4:**
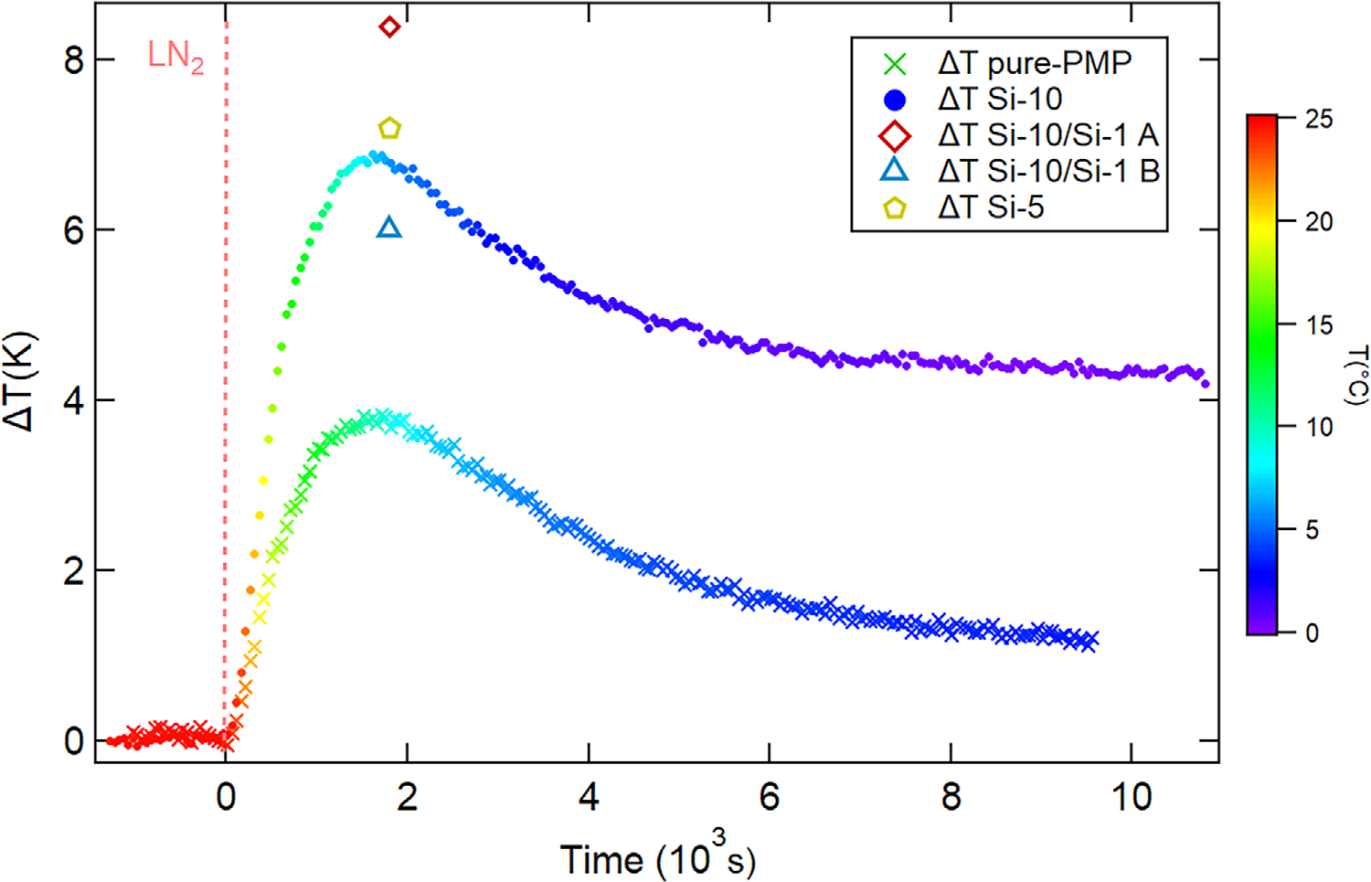
Temperature difference ΔT between the PRDC system and a low‐irradiance dummy, measured under vacuum conditions as a function of time for the polymer‐based samples. The circles correspond to the PMP sample containing 10 µm silica microspheres (Si‐10), while the crosses represents the pure‐PMP sample. The isolated markers at a time delay of approximately $2\cdot 10^{3}$ s correspond to the maximum ΔT achieved for samples Si‐10/Si‐1 A (diamond), Si‐10/Si‐1 B (triangle) and Si‐5 (pentagon). A positive ΔT indicates a temperature of the samples below the one of the low‐irradiance dummy reference. The marker color indicates the absolute PDRC temperature, according to the color scale on the right of the graph.

Before the introduction of the ${\mathrm{LN}}_{2}$, the temperature difference between the PDRC systems and the dummy is constant and near‐zero, as expected for both samples in thermal equilibrium with their environment. After the cold sink starts cooling, a sharp increase in ΔT is observed, implying the PDRC samples have a higher energy dissipation rate with respect to the dummy. As time proceeds, ΔT reaches a maximum for both curves before settling at a value of ΔT ≈ 1.2 K for the pure‐PMP sample and around ΔT ≈ 4.3 K for the silica‐loaded Si‐10 sample. We notice that, starting from room temperature, the Si‐10 system reaches a temperature around 0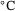, whereas the pure‐PMP system reaches slightly below 5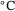.

The energy balance for the PDRCs, in absence of convection, can be written as $P_{rad}\left(T\right)$ + $P_{cond}\left(T\right)=0$, where $P_{rad}\left(T\right)$ ($P_{cond}\left(T\right)$) is the sum of the temperature‐dependent outgoing and incoming radiative (conductive) power, respectively. The equilibrium condition for the PDRCs when exposed to the cold sink is achieved when their temperature allows to balance the above terms: for higher radiative efficiency, such an equilibrium condition occurs at a lower T.

The dynamic of the ΔT curves, that exhibit a maximum a few minutes from the introduction of the ${\mathrm{LN}}_{2}$ sink before settling at a lower equilibrium value, is again due to the temperature dependence of the radiative power, that follows a $T^{4}$ law. Indeed, in the early stages of cooling, the PDRCs are still around room temperature, and their high radiative heat implies a fast cooling. Upon cooling close to 0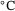, the systems lose about one third of their cooling power, and the relative difference with respect to the dummy sample decreases. The curves show that both polymer‐coated samples (pure‐PMP and Si‐10) have higher radiative power than the dummy, as expected since both systems have absorption/emittance in the mid‐IR. In addition to this, the larger ΔT for the Si‐10 samples demontrates that the silica‐loaded sample exhibits a more efficient radiative emission that the pure‐PMP, showing the clear correlation between the optical response and the radiative efficiency.

## 3 Conclusion

Summarizing, we have reported a combined optical and thermodynamic characterization of systems for passive daytime radiative cooling. The system were fabricated by hot‐pressing a polymeric layer onto an Ag‐covered metal slab. The selected polymer system was either pure poly(4‐methyl‐1‐pentene), or poly(4‐methyl‐1‐pentene) loaded with silica microspheres, in order to improve its emissivity in the mid‐IR spectral range. The silica‐loaded polymer films showed indeed enhanced optical absorption in the 760 and 1250 ${\mathrm{cm}}^{-1}$ spectral window, a range of crucial importance for Earth‐based PDRC, since it corresponds to the atmospheric‐transparency range.

We compared the thermodynamic behavior of either pure‐ or silica‐loaded‐PMP systems under highly controlled and reproducible conditions, showing a clear correlation between the mid‐IR optical absorbance and the cooling efficiency of the devices. We suggest this procedure as an efficient a‐priori evaluation of the suitability of PDRC materials and architectures for their field application.

## 4 Experimental Section

### 4.1 Materials

Poly(4‐methyl‐1‐pentene), PMP, d = 0.835 g ${\mathrm{cm}}^{-3}$, MFR = 26 g (10 min)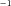 at 260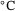 (5.0 kg)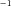 as well as monodisperse, non‐porous, silica microparticles (d = 2.0 g ${\mathrm{cm}}^{-3}$), namely 10 µm (Si10), 5 µm (Si5), and 1 µm (Si1) diameter, were purchased from Merck Life Science Srl (Italy).

### 4.2 Preparation of the PMP‐based Composites

Composites based on PMP matrix containing 6 vol.% of silica microparticles of variable mean diameter (10, 5 and 1 µm) were prepared by using a co‐rotating conical twin‐screw micro mixer‐extruder Xplore MC5 (Xplore Instruments BV, The Netherlands) equipped with a recirculation system allowing the material to circulate under nitrogen purge for 5 min at 270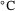 and screw speed of 80 rpm.

### 4.3 Preparation of Stainless Steel Substrate Coated With a Silver Layer

The reflective metallic layer was deposited by physical vapor deposition (PVD) using an electron beam evaporation system manufactured by Kenosistec. The process was carried out under high vacuum conditions, allowing precise control of the film thickness and ensuring high purity of the deposited layers. Due to its high reflectivity in the mid‐ and near‐infrared spectral range and in the visible range, silver was chosen as the metal layer, which is essential for suppressing parasitic absorption and improving radiative cooling performance.

The nominal thickness of the deposited silver layer was 190 nm, which is sufficient to ensure optical opacity and effective reflection of incident radiation. To improve adhesion to the polished stainless‐steel slab and to enhance the stability of the metallic film, two thin titanium dioxide (${\mathrm{TiO}}_{2}$) layers were introduced, each with a thickness of 10 nm, deposited using the same electron beam evaporation technique.

Specifically, a first ${\mathrm{TiO}}_{2}$ layer was deposited directly onto the stainless‐steel substrate, acting as an adhesion layer between the substrate and the silver film. After the deposition of the silver layer, a second ${\mathrm{TiO}}_{2}$ layer was added as a capping layer to protect the silver from oxidation and surface degradation, without significantly affecting its optical properties.

### 4.4 IR‐Emissive Layer: Film Preparation and Deposition by Hot Pressing

Highly transparent films of thickness ≃ 100 microns were achieved by hot‐pressing for 5 min at 250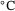 with a pressure of 100 bar the above mentioned extruded PMP‐based composites prior cut in small pellets. The obtained films were adhered onto the substrate coated by the silver layer by hot‐pressing for 2 min at 220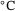 with a pressure of 20 bar, as shown in the Figure 5. The final thickness of the polymer composite film was 30–40 µm. See the Supporting Information for further operational details.

**FIGURE 5:**
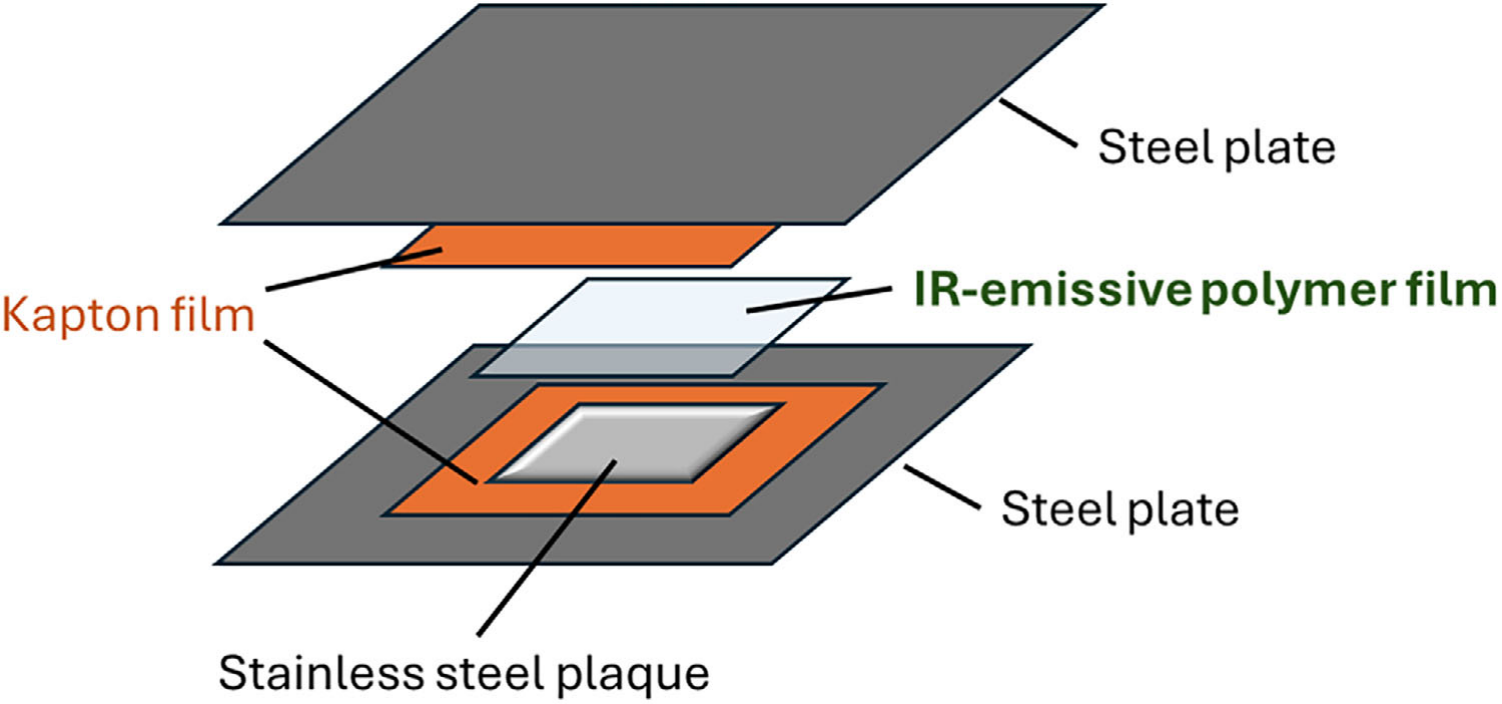
The IR‐emissive polymer film pressed on the substrate between two Kapton films to protect the samples. The steel plates are accessory components of the press.

### 4.5 Thermal Analysis

Thermal behavior of PMP and its composites was investigated by differential scanning calorimetry (DSC) carried out with either with a DSC 821*^e^* or a DSC3+ Mettler–Toledo calorimeter (Mettler–Toledo SpA, Italy). Samples of ca. 10 mg were subjected to a heating‐cooling‐heating cycle under nitrogen atmosphere (flow rate 40 mL $\min^{-1}$) in the range 25–300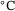, at scan rate 20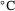 $\min^{-1}$. Heating runs allowed measuring melting temperature ($T_{m}$), and corresponding enthalpy ($\Delta H_{m}$), while during the cooling from the melt, crystallization temperature ($T_{c}$), and corresponding enthalpy ($\Delta H_{c}$) were determined. Thermogravimetric analysis (TGA) was performed on specimens of about 15 mg with a TGA 8000 Perkin–Elmer analyzer (PerkinElmer Scientifica Italia Srl) operating under $N_{2}$ atmosphere from 40 up to 700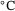 at heating rate of 20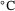 $\min^{-1}$ followed by the introduction of $O_{2}$ into the furnace and temperature increase up to 900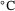 at 20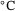 $\min^{-1}$ (gas flow 35 mL $\min^{-1}$).

### 4.6 Morphological Analysis

The morphology of the composites was evaluated using a benchtop scanning electron microscope (SEM) Hitachi TM3000 (Hitachi High Tech, Japan) operating at 15 kV on fragile surfaces obtained by sample fracturing in liquid nitrogen. Specimens were gold sputtered prior observation using a Quorum Q150R ES sputter coater (2M Strumenti Srl, Italy).

### 4.7 Infrared Ellipsometry

Infrared spectroscopic ellipsometry was used to characterize the optical response of the prepared samples in the mid‐infrared region. Measurements were performed using a SENDIRA Infrared Spectroscopic ellipsometer equipped with a Fourier Transform Infrared (FTIR) source (SENTECH Instruments GmbH).

The ellipsometric parameters Ψ and Δ are defined through the relation

(2) $$\tan^{-1}\Psi \cdot e^{i\Delta }=\frac{r_{p}}{r_{s}}$$

where $r_{p}$ ($r_{s}$) is the complex Fresnel reflection coefficient for p‐polarized (s‐polarized) radiation. Ψ and Δ were acquired over a spectral range from 400 to 6000 ${\mathrm{cm}}^{-1}$, which covers the main wavelengths relevant for radiative cooling applications. All measurements were carried out at an angle of incidence of 55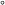.

The ellipsometric spectra were used to obtain qualitative and comparative information on the infrared response of the different samples. These measurements provide an experimental basis for evaluating differences in infrared behavior, which are discussed in detail in the Results and Discussion Section.

### 4.8 Vacuum Chamber Setup for Radiative Cooling Tests

Radiative cooling measurements were performed inside a custom‐designed high‐vacuum chamber equipped with a twin sample holder and a liquid‐nitrogen reservoir placed in front of the sample holder. This chamber serves the purpose of characterizing the materials performances under controlled and reproducible conditions [31, 32]. The vacuum was achieved using a turbomolecular pumping system (Edward TStation 85), allowing a base pressure on the order of 10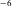 mbar to be reached. The sample holder can host two samples: one of them is a dummy sample (in our case, an Ag‐coated stainless‐steel slab) and the other is the actual PDRC system. The sample holder was designed to minimize parasitic heat conduction to/from the samples. To this end, ceramic tubes were used as thermal insulators between the samples and the metallic holder.

The sample and the dummy were individually connected to two separate thermocouples, and the corresponding temperatures were acquired under identical experimental conditions. The temperature difference between the PDRC sample and the dummy provides us with an estimation of the radiative efficiency of the PDRC, while avoiding spurious artifacts related to the experimental apparatus.

The measurements were taken as follows: the samples were mounted on the holder and inserted in the vacuum chamber; once the target pressure was achieved in the chamber, the reservoir was filled with liquid nitrogen at 77 K, and the temperature acquisition was initiated using the thermocouples connected to a data acquisition system controlled via LabVIEW software. More information and a drawing are reported in the Supporting Information.

## Conflicts of Interest

The author declare no conflicts of interest.

## Supporting information


**Supporting File**: marc70329‐sup‐0001‐SuppMat.pdf.

## Data Availability

The data that support the findings of this study are available from the corresponding author upon reasonable request.
