# Supplementary material for: Correlating Mid‐Infrared Absorption With Radiative Efficiency in Passive Daytime Radiative Cooling Systems
Source: Macromol Rapid Commun. 2026 Jun 17;47(19):e70329. doi: 10.1002/marc.70329 (PMC13639230; doi:10.1002/marc.70329)
Supplement: Supplementary file 1 [file MARC-47-e70329-s001.pdf]

Correlating mid-infrared absorption with radiative efficiency in passive daytime radiative cooling systems

*Emma Spotorno Paola Stagnaro Lucia Conzatti Simona Losio Roberto Utzeri Francesco Bisio**

Emma Spotorno
OptMatLab, Dipartimento di Fisica, Università di Genova, via Dodecaneso 33, 16146 Genova, Italy
Email Address: emma.spotorno@edu.unige.it
Paola Stagnaro, Lucia Conzatti, Roberto Utzeri
CNR-SCITEC, Via de Marini 6, 16149 Genova, Italy
Simona Losio
CNR-SCITEC, Via Corti 12, 20133 Milano, Italy
Francesco Bisio
CNR-SPIN, Corso Perrone 24, 16152 Genova, Italy
Email Address: francesco.bisio@spin.cnr.it

1 Molecular Characterization of PMP by SEC

The weight average molar mass (M_w), number average molar mass (M_n), and molar mass distribution or polydispersity (M_w/M_n) of the commercial PMP (neat-PMP) were determined by high-temperature size exclusion chromatography (HT-SEC) using a Waters GPCV2000 system equipped with a three columns set (Shodex HT 806–805-804) and a Waters 2414 differential refractometer detector Waters 2414. The sample was dissolved in *o*-dichlorobenzene and the measurements carried out at 145 °C and eluent flow rate of 0.8 mL min^{-1}. The universal calibration against polystyrene standards was used. SEC data are reported in Table S1 and in Figure S1.

Table S1: Number-average molar mass (M_n), weight-average molar mass (M_w), and polydispersity index (M_w/M_n) of commercial PMP.

M_n (g mol^{-1})	M_w (g mol^{-1})	M_w/M_n
145900	520600	3.6

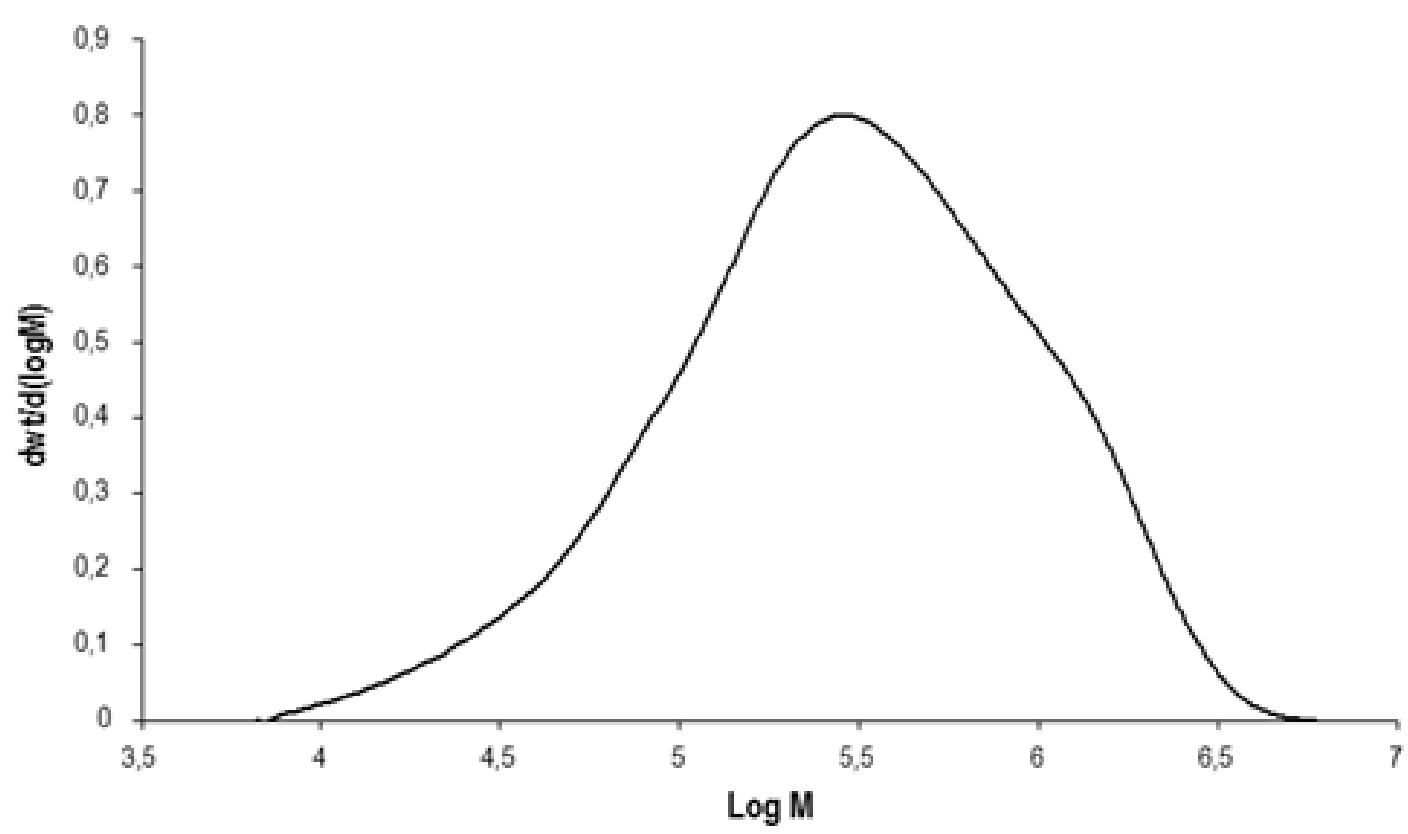

Figure S1: SEC chromatogram of commercial poly(4-methyl-1-pentene) (PMP).

2 IR-emissive PMP-based films by hot pressing

In a first step, PMP-based pellets were hot-pressed at 250 °C under a pressure of 100 bar for 5 min to obtain free-standing films with an area of 4 × 4 cm^2 and a thickness of approximately 100 μm. Subse-

quently, the films were deposited onto polished stainless-steel substrates coated with Ag by a second hot-pressing step performed at 220 °C and 20 bar for 2 min, yielding samples with a final area of 2 × 2 cm^2 and a thickness in the range of 30–40 μm.

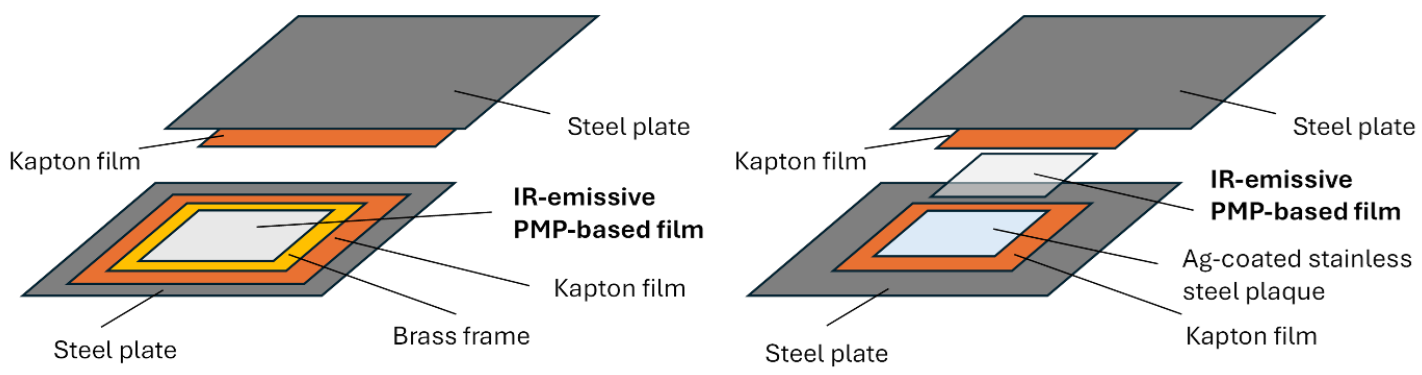

Figure S2: The right side of the Figure shows the schematic of IR-emissive PMP-based film preparation by hot pressing. The left side shows the deposition onto polished stainless steel coated with Ag by hot pressing.

3 Additional SEM Micrographs

Figure S3 and S4 report additional SEM images of the cryo-fractured cross-sections of the PMP-based composites acquired at different magnifications. The micrographs further confirm the homogeneous dispersion of the silica microspheres within the polymer matrix. No significant aggregation phenomena are observed, indicating good compatibility between the silica microspheres and the PMP matrix.

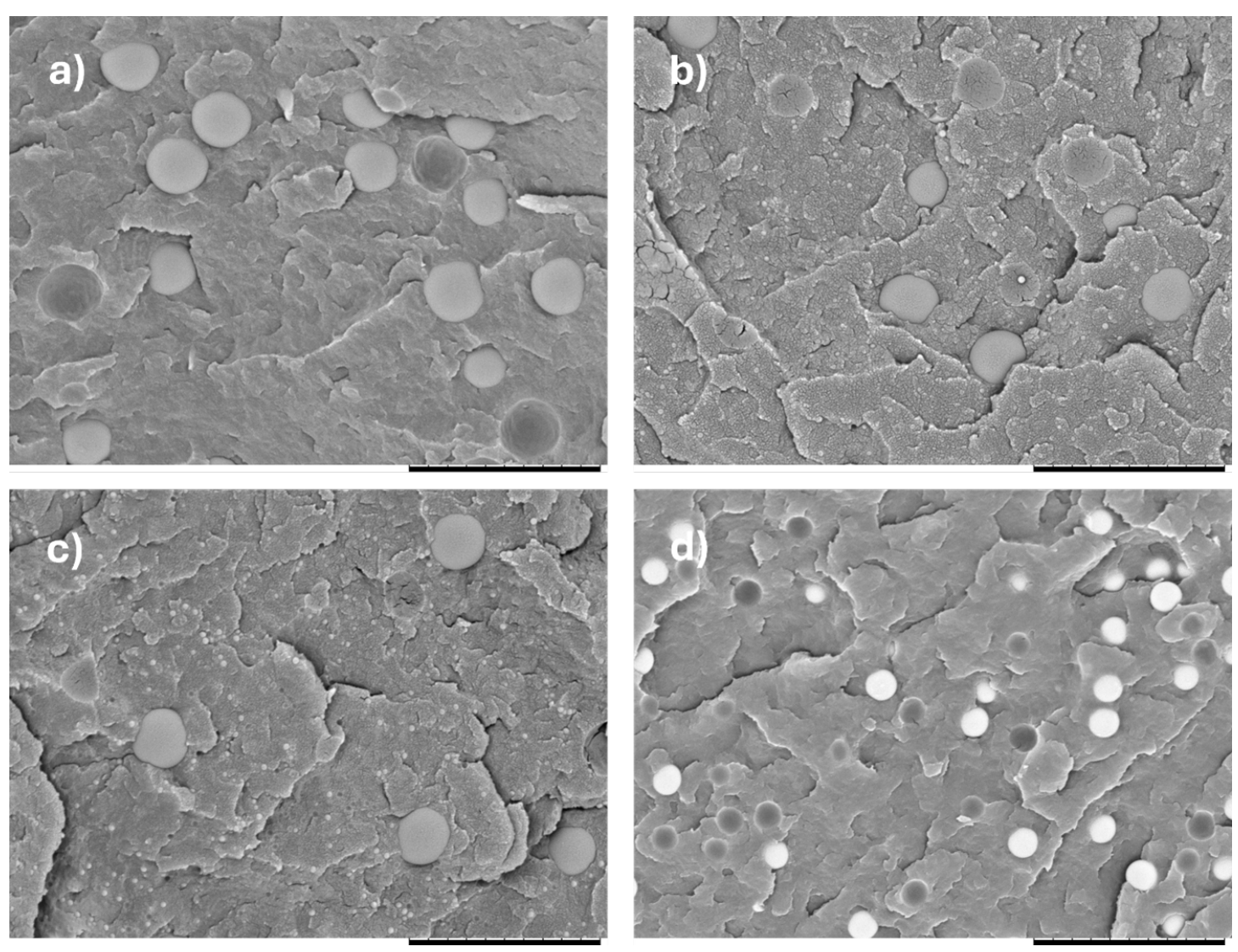

Figure S3: SEM images of the PMP-based composite samples with different silica microsphere compositions. (a) Si-10; (b) Si-10/Si-1 A; (c) Si-10/Si-1 B; (d) Si-5. (SCALE BAR: 30 μm)

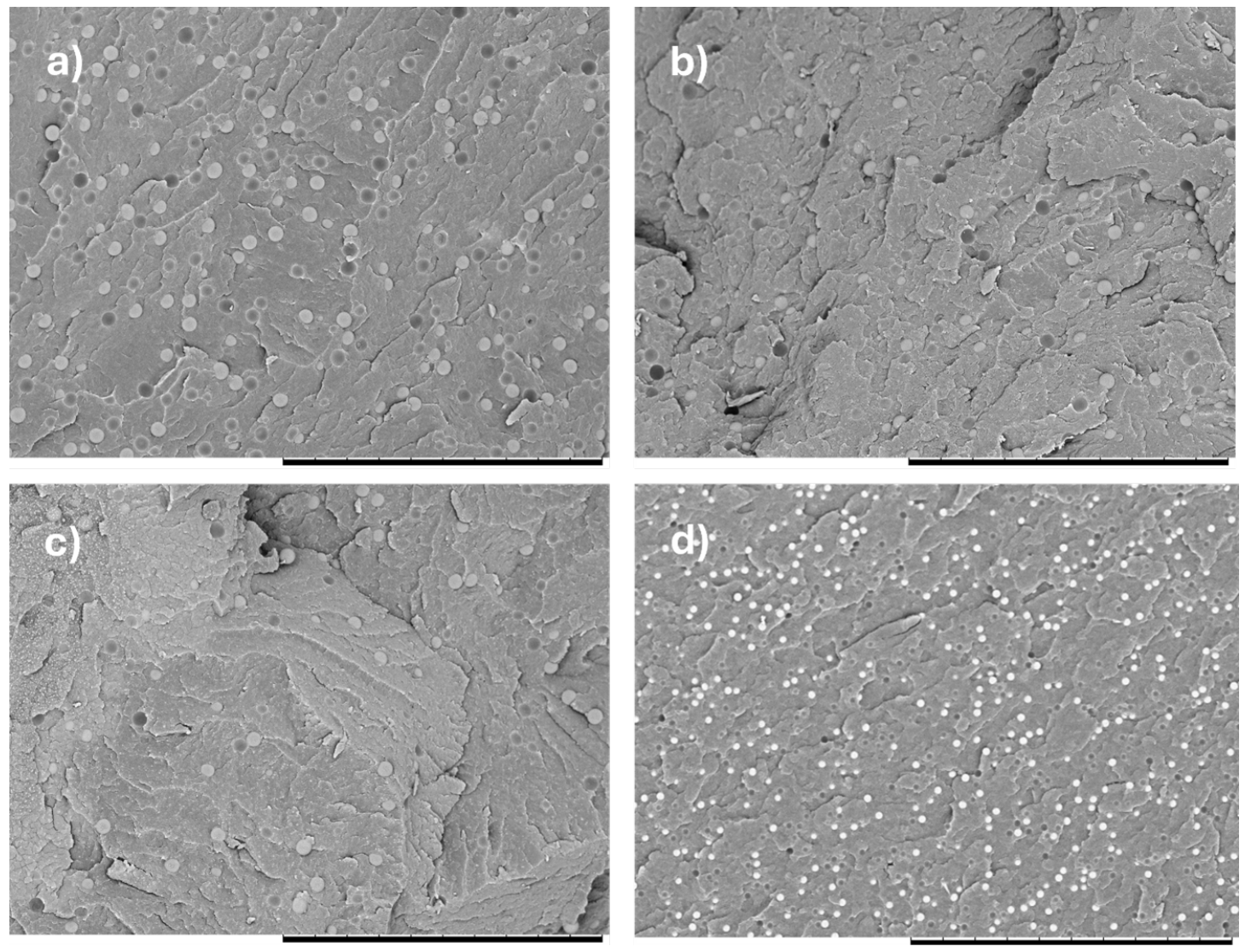

Figure S4: SEM images of the PMP-based composite samples with different silica microsphere compositions. (a) Si-10; (b) Si-10/Si-1 A; (c) Si-10/Si-1 B; (d) Si-5. (SCALE BAR: 200μ m)

4 Correlation between dielectric function and ellipsometric parameters

In order to establish a correlation between the Ψ, Δ spectra reported in the main text and the absorbance of the polymer layer, we need to develop a simplified optical model of the system under consideration. This simplified optical model was based on Voigt oscillator combined with a Cauchy dispersion term, and allowed to study the relationship between absorption properties and the ellipsometric response in the infrared region. The aim of this modeling was not to provide an absolute measure of the emissivity of the material, but rather to justify the semi-quantitative use of the Ψ spectral ellipsometric parameter as indicator of absorbance in the polymer layer. Voigt line shapes were selected to represent the infrared vibrational bands of the polymeric materials.
We started by constructing a model consisting of a 40 μm-thick transparent layer, whose optical response is well represented by a Cauchy-type optical dispersion, laid on top of an optically-thick Ag film. In the mid-IR region, the Ψ of this system reads 45° (dashed fuchsia line in Figure S5).
Following this, we introduced an optical absorption in the transparent film, having the functional form of a complex Voigt oscillator. Under these conditions, the imaginary part ε_2 of the dielectric function of the 40 μm-thick is represented by the solid blue-green line in the bottom panel of Figure S5, and the corresponding Ψ by the dashed blue-green line in the top panel. this clearly shows that spectral dips in Ψ below the 45° level are indicative of optical absorption in the otherwise transparent film.

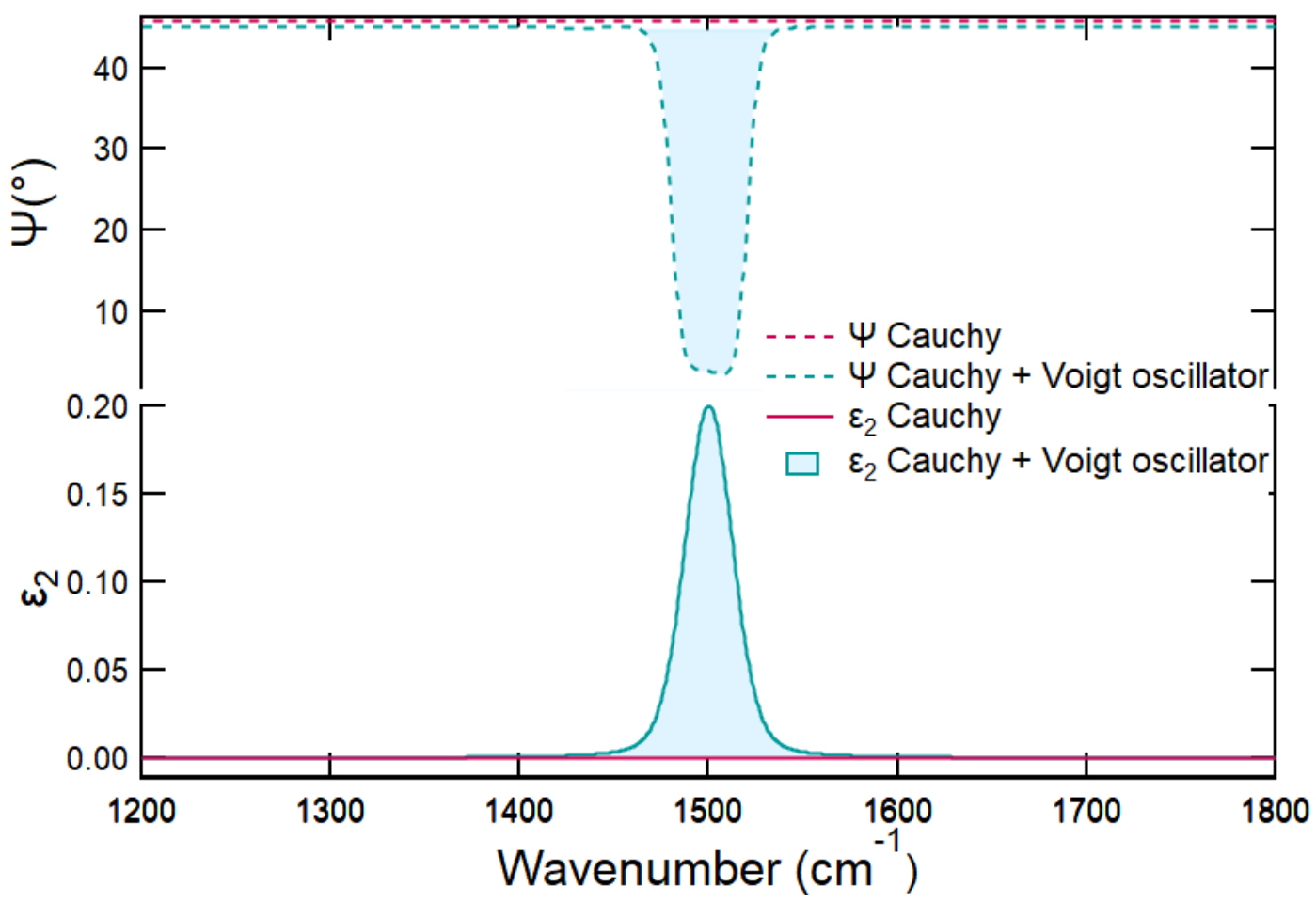

Figure S5: The upper panel shows the Ψ spectra for the two models. The dashed blue–green line corresponds to the Cauchy dispersion term combined with a Voigt oscillator, while the dashed fuchsia line represents the Cauchy-type dispersion model.The lower panel displays the imaginary part of the dielectric function, ϵ_2, where the solid blue–green line refers to the Cauchy term with the Voigt oscillator and the solid fuchsia line corresponds to the Cauchy term alone. The areas under the Ψ and ϵ_2 curves are highlighted in light blue.

We then proceeded to establish the relation between ε_2 and Ψ by keeping fixed the resonance frequency and the spectral broadening of the complex Voigt oscillator and gradually increasing its magnitude, hence the degree of optical absorption within the transparent layer. The relation between the total absorbance, proportional to the integral of the imaginary part of the dielectric function ϵ_2 of the polymer layer, and the magnitude of the dip in Ψ is not linear, as shown in Figure S6.

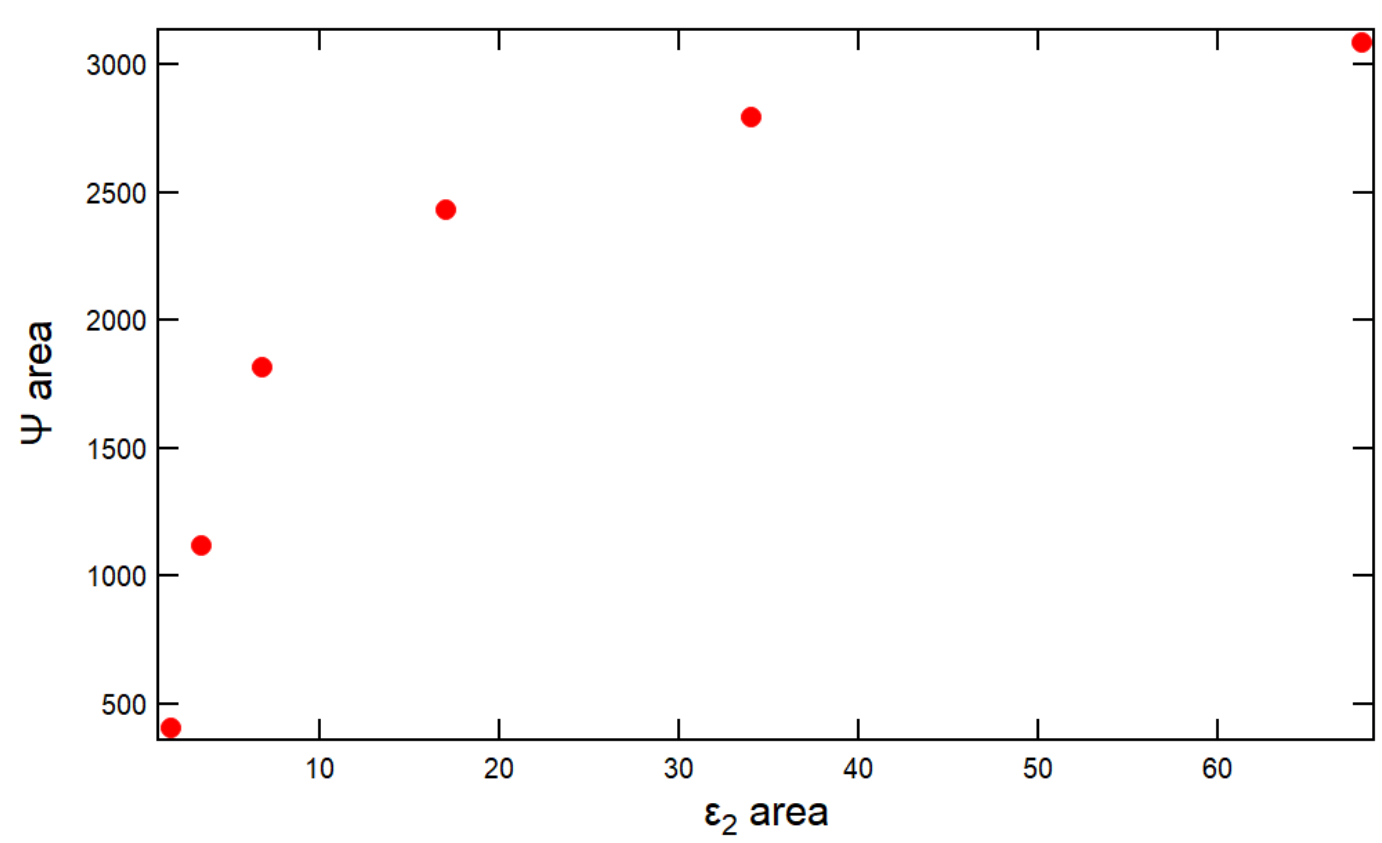

Figure S6: Relation between the integral of the imaginary part, ϵ_2, of the dielectric function and the total absorbance derived from the Ψ spectrum. The values in the Figure S6 were obtained gradually increasing the Voigt oscillator amplitude.

In particular, this simplified treatment shows that the integrated magnitude of the Ψ dips follows linearly the integral of the imaginary part of the dielectric function ϵ_2 for small values of absorbance and saturates for larger values. Nonetheless, the presence and magnitude of the spectral dips in the Ψ data represents a first-order indication of the presence and relevance of optical absorption in the polymer layer.

5 Optical response of silica-loaded PMP films

The Figure S7 shows the IR ellipsometric parameters Ψ (solid curves) and Δ (dashed curves) for the additional SiO_2-loaded PMP samples not discussed in the Results and Discussion Section. Si-10/Si-1 A sample contains a distribution of silica microspheres with a 75:25 volume ratio of 10 μm and 1 μm diameter, Si-10/Si-1 B sample contains a distribution of silica microspheres with a 50:50 volume ratio

of 10 μm and 1 μm diameter, while Si-5 sample is loaded with 5 μm silica microspheres. Consistently with the behaviour reported in the main text, the incorporation of silica microspheres enhances their absorbance in the atmospheric transparency window (670–1250 cm^{-1}). Moreover, spectral features are systematically observed around 3000 cm^{-1}, associated with intrinsic vibrational contributions of the polymer backbone and side groups, in agreement with the assignments discussed in the main text.

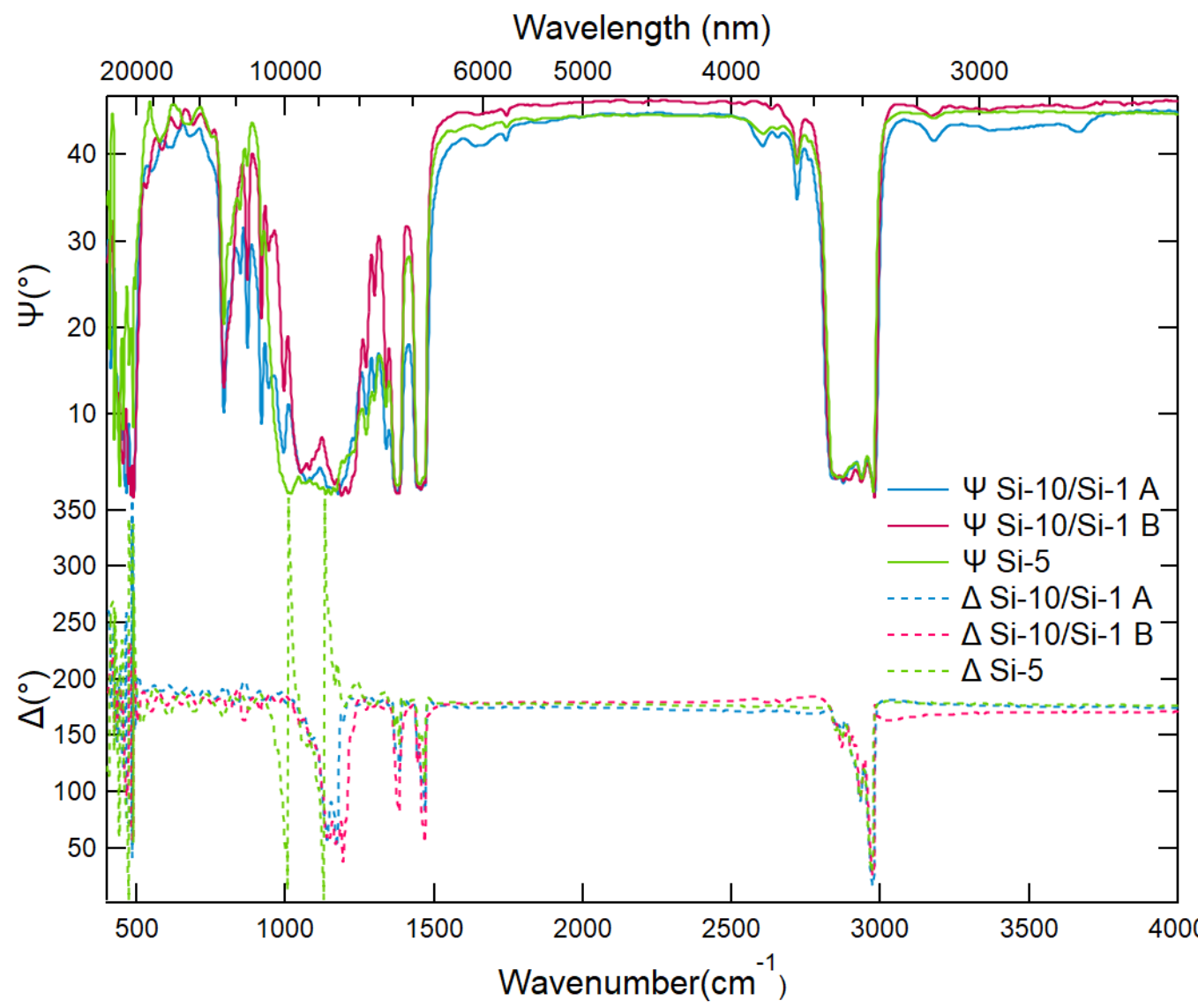

Figure S7: IR ellipsometric parameters Ψ (solid lines) and Δ (dashed lines) for Si-10/Si-1 A sample (light-blue line), Si-10/Si-1 B sample (fuchsia line) and the Si-5 sample (green line).

6 Vacuum Chamber SetUp

Figure S8 shows a schematic representation of the experimental configuration adopted for the radiative cooling measurements. The figure is reported here to highlight the geometrical arrangement of the samples, which is relevant for the interpretation of the temperature differences discussed.

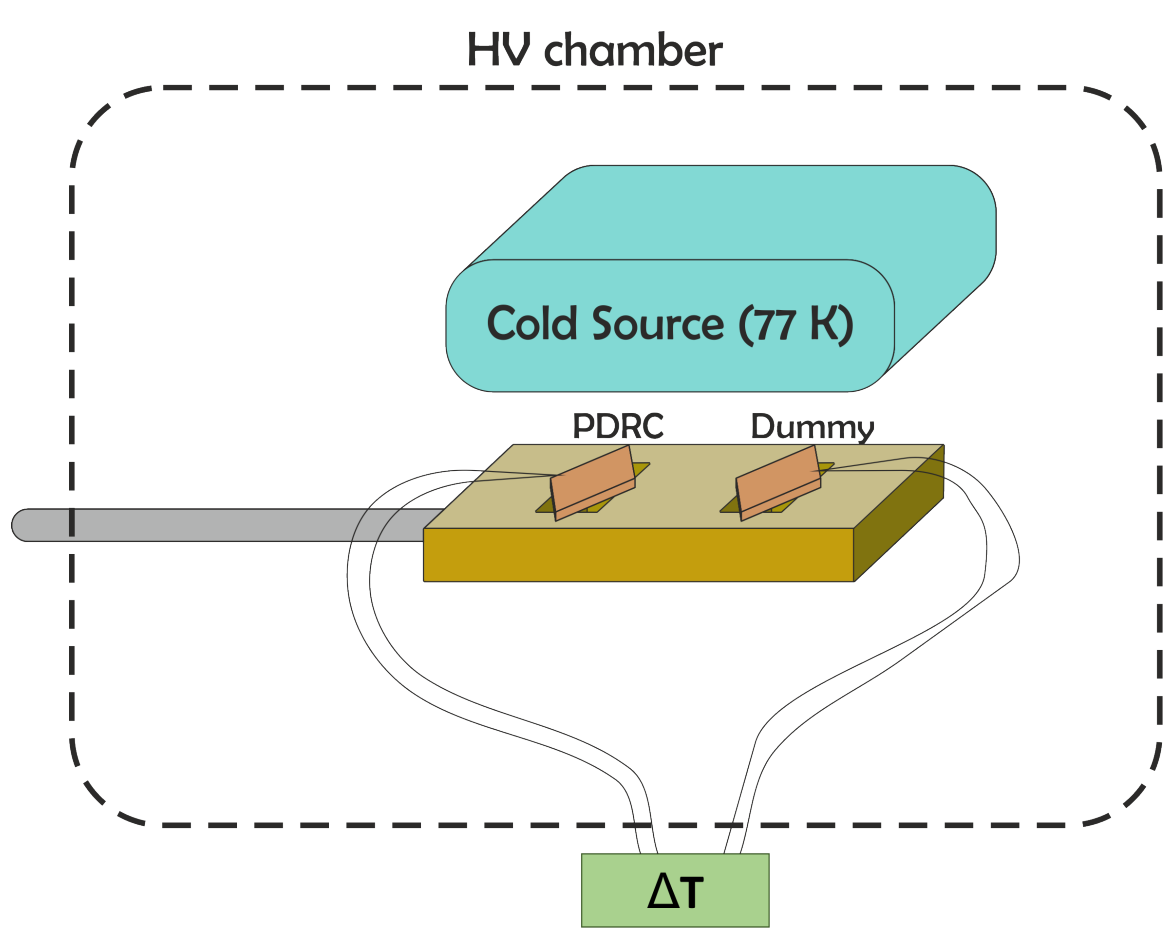

Figure S8: Vacuum chamber with the cold source (LN_2) and the sample-holder with thermocouples. Set-up for radiative cooling measurements.

The experiments were carried out under high-vacuum conditions in order to suppress convective and conductive heat transfer through the surrounding environment. Under these conditions, the thermal exchange between the samples and the liquid-nitrogen reservoir is dominated by radiative heat transfer. A dummy reference sample and a polymer-based radiative cooling sample were mounted on the same sample holder and measured simultaneously. The sample holder and its supports were designed to mini-

mize parasitic heat conduction, so that differences in temperature evolution can be primarily attributed to the radiative properties of the samples.
